# Does it make sense to target one tumor cell chemotactic factor or its receptor when several chemotactic axes are involved in metastasis of the same cancer?

**DOI:** 10.1186/s40169-016-0113-6

**Published:** 2016-08-10

**Authors:** Mariusz Z. Ratajczak, Malwina Suszynska, Magda Kucia

**Affiliations:** 1 Stem Cell Institute at James Graham Brown Cancer Center, University of Louisville, 500 S. Floyd Street, Rm. 107, Louisville, KY 40202 USA; 2 Department of Regenerative Medicine, Medical University of Warsaw, Warsaw, Poland

**Keywords:** Cancer metastasis, Pro-metastatic microenvironment, SDF-1, S1P, C1P, HGF

## Abstract

The major problem with cancer progression and anti-cancer therapy is the inherent ability of cancer cells to migrate and establish distant metastases. This ability to metastasize correlates with the presence in a growing tumor of cells with a more malignant phenotype, which express certain cancer stem cell markers. The propensity of malignant cells to migrate and their resistance to radio-chemotherapy somewhat mimics the properties of normal developmentally early stem cells that migrate during organogenesis in the developing embryo. In the past, several factors, including cell migration-promoting cytokines, chemokines, growth factors, bioactive lipids, extracellular nucleotides, and even H^+^ ions, were found to influence the metastasis of cancer cells. This plethora of pro-migratory factors demonstrates the existence of significant redundancy in the chemoattractants for cancer cells. In spite of this obvious fact, significant research effort has been dedicated to demonstrating the crucial involvement of particular pro-metastatic factor–receptor axes and the development of new drugs targeting one receptor or one chemoattractant. Based on our own experience working with a model of metastatic rhabdomyosarcoma as well as the work of others, in this review we conclude that targeting a single receptor–ligand pro-metastatic axis will not effectively prevent metastasis and that we should seek other more effective therapeutic options.

## Introduction

**Fig. 1:**
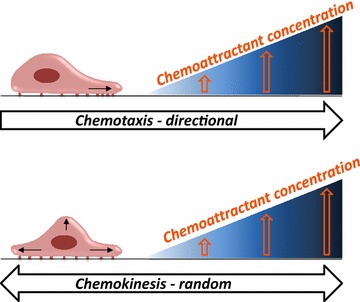
The difference between chemotaxis and chemokinesis. Cells may respond to a pro-migratory factor in two different ways: by directed movement, in the process *chemotaxis*, or by random multidirectional movement, in the process of *chemokinesis*. Both mechanisms may be involved in egress of cancer cells from the primary tumor

Metastasis is responsible for more than 90 % of cancer-associated mortality, and preventing its occurrence is a therapeutic priority in clinical oncology [1]. Several factors have been identified that induce the migration of cancer cells, both in the process of directional cell migration known as chemotaxis [2] and the random multidirectional migration termed chemokinesis [2]. Both of these processes (Fig. 1) lead to egress of cancer cells from the primary tumor, relocation to distant sites, and the establishment of metastases. Usually, chemotaxis and chemokinesis together play a role in the motility of cancer cells. However, depending on the type of a given tumor, various chemotactic factors may promote more of one or the other cell-trafficking mechanism.

The list of candidate metastatic factors for cancer cells is very long and includes cell migration-promoting chemokines (e.g., stromal-derived factor 1, SDF-1), growth factors (e.g., hepatocyte growth factor/scatter factor, HGF/SF), bioactive lipids (e.g., sphingosine-1-phosphate, S1P; ceramide-1-phosphate, C1P), extracellular nucleotides (e.g., ATP, UTP), and even H^+^ ions [3–10]. The migration of cancer cells may also be affected by certain hormones (e.g., follicle-stimulating hormone, FSH; luteinizing hormone, LH), cleavage fragments of the complement cascade (C3 and C5 cleavage fragments; C3a and C5a, respectively), components of the coagulation cascade (e.g., thrombin), and certain danger-associated molecular pattern molecules (DAMPs; e.g., S100 proteins) [11–16].

Pro-metastatic factors activate various specific, corresponding types of receptors, including cytokine receptors, tyrosine kinase receptors, and G protein-coupled receptors. Signals transduced from these receptors activate similar signaling pathways involved in the regulation of cell migration or adhesion and affect elements of the intracellular cytoskeleton [17–19].

The redundancy of factors and receptors involved in migration of cells in the same type of cancer poses an important question: Is it reasonable to target particular pro-migratory axes when several other pro-metastatic axes exist for a given tumor cell? Moreover, in most of the published reports demonstrating migration, “supraphysiological concentrations” of pro-metastatic factors were employed at doses not encountered in normal tissues and that may not be relevant to clinical situations. In addition, the responsiveness of primary tumor cells may change over time as a malignancy progresses and could be affected by several additional clinical problems that emerge in patients, such as infections or organ failure.

In this review we will summarize several years of experience in identifying and blocking crucial pro-metastatic axes involved in the metastasis of human rhabdomyosarcoma (RMS) cells [6, 12, 20–25]. Our observations, obtained with an RMS cell metastasis model, are also relevant to other types of malignancies, as significant redundancy in pro-metastatic ligand–receptor axes exists for almost all tumor types studied so far.

## Rhabdomyosarcoma as a model to study cancer metastasis

Rhabdomyosarcoma (RMS) is the most common soft-tissue sarcoma of adolescence and childhood and reportedly accounts for 5 % of all malignant tumors in patients under 15 years of age [26]. Two major histological subtypes have been described: alveolar rhabdomyosarcoma (ARMS) and embryonal rhabdomyosarcoma (ERMS) [27]. ARMS is associated with more aggressive behavior and a worse prognosis than ERMS [28]. Together with neuroblastoma, nephroblastoma, and Ewing’s sarcoma, RMS belongs in the family of so-called “small round blue tumor cells”, which often infiltrate bone marrow (BM). These tumor cells on BM smears are sometimes misdiagnosed as acute leukemia cells [29, 30].

The two types of RMS show differences at the molecular level. ARMS is characterized by the translocation (2;13)(q35;q14) in 70 % of cases and the variant translocation (1;13)(p36;q14) in a smaller percentage of cases [31, 32]. These translocations disrupt the *PAX3* and *PAX7* genes on chromosomes 2 and 1, respectively, and the *FOXO1* gene on chromosome 13, which leads to the generation of *PAX3*–*FOXO1* and *PAX7*–*FOXO1* fusion genes. PAX3–FOXO1 and PAX7–FOXO1 fusion proteins have enhanced transcriptional activity compared with wild type PAX3 and PAX7 and are postulated to play a role in cell survival and dysregulation of the cell cycle in ARMS [31]. Since there are also ARMS cases that are fusion-negative and have a better outcome than fusion-positive cases, it was more recently recommended that RMS should be classified into fusion-positive (*PAX3*–*FOXO1* and *PAX7*–*FOXO1*) and fusion-negative tumors [7]. In our experiments over the past 15 years to study RMS metastasis, we have employed several human RMS cell lines, including both fusion-positive (e.g., RH28, RH30, RH41) and fusion-negative (e.g., JR, RD, RH18, RH36, SMS-CTR) tumor cell lines [8, 20, 21]. Some of our results were subsequently verified in primary RMS patient tissue samples [25, 33].

However a lot of progress has been made to understand pathogenesis of RMS, the origin of cells that gives rise in skeletal muscle tissue to this malignancy is still under debate. It has been proposed that, while low-passage mesenchymal stem cells (MSCs) can generate ARMS, low-passage myoblasts can form ERMS [34–36]. On the other hand, RMS cells express several cancer testis antigens (CTAs), which are characteristic of germline-derived cells [37–41]. This observation makes a somewhat hypothetical connection to a concept presented 150 years ago by Rudolf Virchow [42] and Julius Conheim [43], who proposed the “embryonic rest hypothesis of cancer development” [44]. According to this hypothesis, certain malignancies may develop from dormant embryonic or germ cells residing in adult tissues [44]. In this context, small round blue cell tumors, (e.g., RMS) that express several CTA antigens are potential candidates to form such malignancies. This hypothesis, however, requires further study. In any case, RMS cell lines, which are endowed with migratory potential, are a convenient model for studying cancer metastasis.

## Assays with which to study the metastasis of cancer cells

**Fig. 2:**
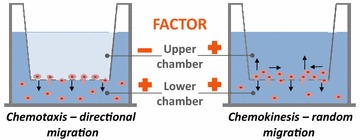
In vitro Transwell migration assay. Cells to be tested are placed in the *upper chamber*, and the migration-promoting factor to be tested for* chemotaxis* is placed in the *lower chamber*. If the factor is to be tested for* chemokinesis*, it is added at the same time to both *upper* and *lower chambers*. Cells that migrate to the *lower chamber* are counted and compared with cells that had migrated in medium without the pro-migratory factor (the control Transwell inserts)

The metastatic potential of tumor cells can be studied by employing several complementary assays. The most convenient is the Transwell migration assay, which employs two chambers separated by a porous membrane [45]. The cells to be tested are loaded into the upper chamber, and the chemoattractant is added to the lower chamber. The readout in this assay is the number of cells that migrate from the upper to the lower chamber in response to a chemotactic factor, which is a process known as chemotaxis. This system also allows us to measure random cell migration, which is a process known as chemokinesis [45]. In order to study chemokinesis, a pro-metastatic factor is added to both the lower and upper chambers, and chemokinesis is said to occur when a gradient is missing between chambers and cells still migrate to the lower chamber from the upper chamber (Fig. 2).

**Fig. 3:**
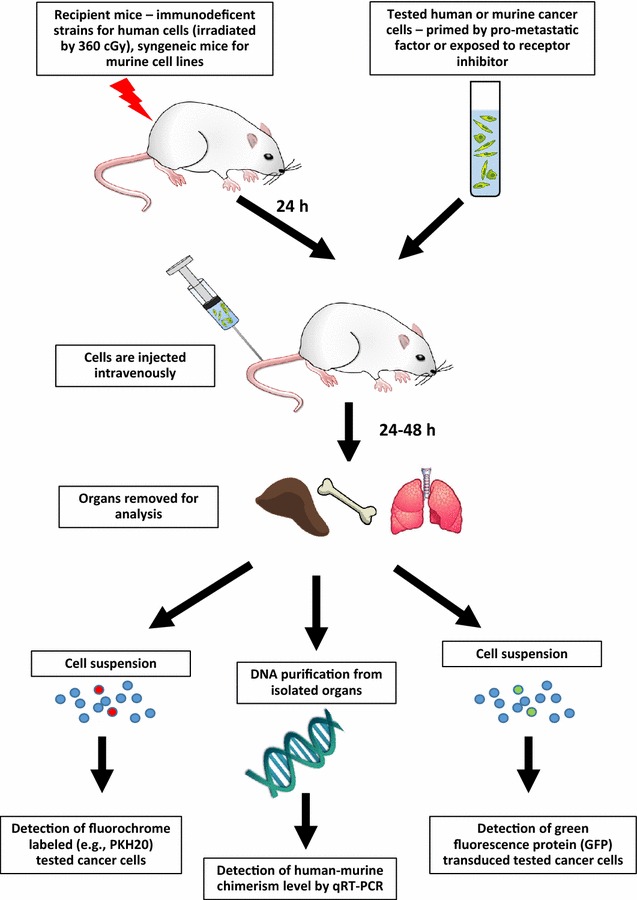
In vivo seeding efficiency assay for human cells. Human cells exposed ex vivo (primed) to a pro-metastatic factor or a receptor blocking agent are subsequently injected i.v. into immunodeficient mice. Mice can be additionally irradiated with 360 cGy. The number of human cells can be detected in murine organs by FACS (after labelling cells with fluorochrome or transducing with GFP) or by detecting the level of human DNA in murine organs

In contrast to in vitro Transwell migration, another relatively easy in vivo assay with which to study the metastasis of human cancer cells is the “cancer cell seeding assay” developed by us [8, 20–22] (Fig. 3). This assay is based on intravenous injection of tumor cells into immunodeficient mice; 24–48 h later, the organs are extracted to detect the presence of human cells. Human cells in murine tissues can be detected directly by FACS if the injected cells carry fluorescent markers (e.g., transduced with the gene encoding GFP protein or labeled ex vivo with PKH26) or indirectly by detecting human DNA in murine tissues using RQ-PCR (e.g., to detect human DNA specific for α satellite sequences) and comparing the amplification result to a standard curve established by mixing human and murine cells in different ratios [8, 20]. From the percentage of human DNA present in DNA extracts, we can estimate how many human cells were present in a given organ using this standard curve [8, 24]. Before injection into experimental animals, the cancer cells may be stimulated with pro-metastatic factors or exposed to the inhibitor of their corresponding receptors.

By employing this in vitro Transwell assay and the in vivo cancer cell seeding efficiency assay, it is possible, in a relatively easy way, to study the contribution of several potential pro-metastatic factor–receptor axes to cancer metastasis and to test the efficacy of various anti-metastatic strategies [8, 21, 22, 33].

## “The never-ending story” of pro-metastatic factors for RMS cells

In the past 15 years we have identified several factors involved in directing the migration of RMS cells and thus potentially directing metastasis of this tumor. The first factors that we studied were cytokines with chemotactic activity, known as chemokines [6, 9, 20–22]. Chemokines regulate the migration of several types of normal cells, activate seven-transmembrane-domain G protein-coupled receptors, and it is not surprising that they also chemoattract cancer cells [18, 23, 36, 46–48]. For example, we demonstrated that SDF-1, by engaging both CXCR4 and CXCR7 seven-transmembrane-domain receptors, promotes migration of RMS cells and could be responsible for their metastasis to BM [6, 22]. Specifically, we showed that RMS cells respond robustly to gradients of SDF-1 employed at high concentrations, and this migration was inhibited by blocking CXCR4 with small-molecule antagonists [6]. Later on, when a new ligand for CXCR4, the chemokine macrophage migration inhibitory factor (MIF), had been described [49], we also confirmed that it may direct migration of CXCR4^+^ RMS cells [21]. Since RMS cells express CXCR7, they may also respond to another chemokine, interferon-inducible T cell alpha chemoattractant (I-TAC) [22]. The role of chemokines in regulating the biology of RMS cells is even more complicated, as RMS cells may secrete interleukin 8 (IL-8). Since they do not express the corresponding receptors (CXCR1 and CXCR2), IL-8 secreted by RMS cells exerts paracrine effects on the surrounding microenvironment and stimulates tumor angiogenesis [50].

RMS cells also respond to several growth factors that engage receptors with intrinsic tyrosine kinase activity [18, 51]. It has been reported that insulin-like growth factor 1 and 2 are not only RMS growth-promoting factors but are also potent chemotactic factors for these cells [18, 52–54]. In our own work we also confirmed that hepatocyte growth factor/scatter factor (HGF/SF) promotes migration and adhesion of RMS cells by engaging the c-Met receptor [20].

Another group of factors that may direct migration of RMS cells are cytokines, and our recent research demonstrated the involvement of erythropoietin in enhancing the pro-metastatic potential of this tumor [55]. Erythropoietin is very often employed in patients to ameliorate chemotherapy-induced anemia [56]. Therefore, erythropoietin supplementation in RMS patients may have the unwanted side effect of stimulating tumor progression.

In addition to peptide-based factors, such as cytokines, chemokines, and growth factors, another potent class of pro-metastatic factors for RMS cells that we have identified is bioactive lipids. In our recent work we have demonstrated that the pro-metastatic potential of RMS cells is enhanced by the presence of sphingosine-1-phosphate (S1P), ceramide-1-phosphate (C1P), lysophosphatidylcholine (LPC), and its derivative, lysophosphatidic acid (LPA) [25]. All these bioactive lipids strongly enhance motility and adhesion of human RMS cells, and, more importantly, these metastatic-associated phenomena were observed at physiological concentrations of these lipids that naturally occur in biological fluids [25].

Moreover, a novel class of factors that we identified that may enhance the migration of RMS cells is gonadal and pituitary sex hormones (SexHs) [55]. SexHs are involved in skeletal muscle development and regeneration, and we found that follicle-stimulating hormone (FSH) and luteinizing hormone (LH) receptors are expressed in established human RMS cell lines as well as in primary tumor samples isolated from RMS patients. We also found that human RMS cell lines responded both to pituitary and gonadal SexH stimulation by enhanced proliferation, chemotaxis, and cell adhesion [55]. The expression of functional SexHs by RMS cells suggests, as mentioned above, their developmental relationship with certain developmentally early stem cells deposited in adult tissues [57, 58].

Finally, metastasis and migration of RMS cells are also affected by several other factors, such as extracellular microvesicles (ExMVs) [59, 60], thrombin [12], and even extracellular nucleotides (e.g., ATP, UTP) [24]. The list of these factors is still open, and new candidates are being identified.

## Other strategies to inhibit the metastasis of cancer cells

Based on our results for a model of RMS metastasis, we conclude that there are multiple redundant pro-metastatic axes for this tumor. Therefore inhibition of one of these axes will not prevent pro-metastatic responsiveness of the cells to other axes. Instead, most ideal anti-metastatic treatment should target common mechanisms involved in the metastatic process.

One of these possibilities is to target signaling pathways involved in cell migration such as intracellular kinases that are known to promote this process (e.g., p42/44 MAPK, AKT, or PKC). However, since these signaling kinases are involved in the regulation of many physiological processes, it would be difficult to target them without unpredictable side effects.

Another potential strategy would be to enhance the expression of certain stress-specific pathways that inhibit cell migration. One such strategy that we recently identified is to upregulate heme oxygenase 1 (HO-1) in cancer cells (manuscript in preparation). Small-molecule inducers of this stress-induced enzyme are available, and our preliminary results in several tumor models demonstrate the efficacy of such treatment in inhibiting the spread of cancer cells [61, 62]. However, in parallel we have to take into consideration other potential pleiotropic effects of HO-1 on tumor cells.

Finally, we have proposed that a pro-metastatic microenvironment may be induced in healthy tissues in response to radio-chemotherapy [63, 64]. While there are several well-known side effects of chemotherapy and radiotherapy that are mainly related to toxicity and the impaired function of several organs, the induction of a pro-metastatic microenvironment is still, surprisingly, not widely acknowledged [63, 64]. We proposed that toxic damage in various organs leads to upregulation in “bystander” tissues of several chemotactic factors, which attract circulating stem cells for regeneration but unfortunately also provide chemotactic signals to attract cancer cells that survived the initial treatment [63]. This mechanism may play an important role in the metastasis of cancer cells to organs such as bones, lungs, and liver, which are highly susceptible to chemotherapeutic agents as well as ionizing irradiation. We have demonstrated that this side effect of radio-chemotherapy can be ameliorated by administration of non-steroid anti-inflammatory drugs (e.g., ibuprofen) or steroids at the time of administration of radiochemotherapeutic treatment [63]. This strategy may effectively ameliorate collateral induction of pro-metastatic factors in various organs and tissue. Based on our experimental data these new potential therapeutic strategies shall be tested in the clinical settings.

## Conclusions

As mentioned in introduction metastasis is responsible for more than 90 % of cancer-associated mortality and therefore the clinical need to prevent or target metastasis is one of the therapeutic priorities in clinical oncology. Our long-standing studies on the mechanisms involved in cancer metastasis by employing RMS cells as a model system clearly show that targeting a single receptor–ligand axis may slow down but will not prevent cancer cells from undergoing metastasis, as several redundant pro-metastatic receptor–ligand axes exist. Moreover, based on the literature and taking into consideration that multiple pro-metastatic factors have also been identified for other types of malignancies, the general conclusions of this review apply also to other tumors. Therefore, there is an urgent need to develop more efficient anti-metastatic therapies that will simultaneously target the response of cancer cells to all pro-metastatic factors (e.g., by intracellular upregulation of HO-1) or even to employ as a prophylactic treatment drugs (e.g., steroids or anti-inflammatory drugs) that prevent induction of a pro-metastatic microenvironment induced in various organs after radio-chemotherapy. These new potential anti-metastatic strategies could be combined with surgical treatment and/or cancer immunotherapy. A variety of novel surgical approaches as well as strategies to stimulate the immune system to destroy growing tumors are available including T-cell adoptive transfer combined with inteleukin-2 therapy, genetically engineered T cells specialized to recognize tumor antigens or autologous immune enhancement therapy using patient’s own peripheral blood-derived NK cells or other relevant immune cells [65–67].
